# Mental Health Disorders During the COVID-19 Outbreak in Cyprus

**DOI:** 10.25122/jml-2020-0114

**Published:** 2020

**Authors:** Neophytos Stylianou, Gregoria Samouti, George Samoutis

**Affiliations:** International Institute for Compassionate Care, Nicosia, Cyprus

**Keywords:** COVID-19, anxiety, depressive symptoms, Cyprus

## Abstract

Cyprus has been affected by COVID-19 since March 2019. With a case fatality rate of 2.6% (until June 2020) and the social isolation measures enforced on the population, the population’s mental health has been affected. We aimed to assess the mental health burden of the Cypriot population during the outbreak and to explore the potential influence factors.

Using a web-based cross-sectional survey, we collected data from 216 volunteers regarding demographic data, COVID-19-related knowledge, generalized anxiety disorder (GAD), and major depressive symptoms. The overall prevalence of GAD and major depressive symptoms of the public were 13.89% and 8.33%, respectively. No demographic had shown any statistical significance with GAD. The younger age group of the study showed a statistically significant association with major depressive symptoms when compared to the adult population in both univariate and multivariable analyses.

Our study identified a mental health burden of the Cypriot population, especially the younger age groups. As part of the preparedness for situations as the one we are experiencing and the future impact the pandemic may have on society, interventions should be focused on vulnerable groups of the population to alleviate the psychosocial effects.

## Introduction

An unknown cause of pneumonia was reported to the World Health Organization (WHO) from China on December 31, 2019. After a month, the outbreak has spread to more than 18 countries, and it was declared a Public Health Emergency of International Concern [1]. On March 11, 2020, the WHO declared the disease a pandemic [2]. As of March 7, Cyprus was also on the COVID-19 map. Since then and until May 19, there were 918 confirmed cases and 24 COVID-19-related deaths. This gives a case fatality ratio (CFR) of 2.6% [3].

The main measures to prevent the spread of transmission that the Cypriot government took were the prohibition of any citizen to enter the country, closure of shopping malls and other shops such as barbers, hairdressers and beauty centers. Measures were enforced on the population, such as the prohibition of unnecessary movement, and no movement between 06:00 and 21:00 [4]. The movement of population was only allowed for work purposes, although most workplaces required their employees to work from home when possible, and only one other outing was allowed for purposes such as physical activity, visits to doctors/pharmacists, grocery shopping and others.

The virus’s high spreadability, the lack of a targeted treatment, the absence of a vaccine, the strict measures enforced by governments and the unregulated bombardment of information through social media and the Internet have caused universal anxiety and distress. In addition to physiological damage that the virus can cause to the human body, it has also created a serious impact on the public’s mental health. Similar to population behaviors faced by many countries, the Cypriot population has also shown anxiety-related behaviors, causing a significant shortage of medical masks and alcohol-based disinfectants across the country. The WHO acknowledged that these psychological responses are natural and that the implemented prevention measures will have an effect on the population [5].

Using a web-based cross-sectional study, we aimed to assess the effect the disease and the associated “infodemic” have on the Cypriot population’s mental health during the outbreak and to explore potential influence factors.

## Material and Methods

### Data Collection

Participants answered the questionnaires anonymously on the Internet from 10/04/2020 to 25/05/2020.

The survey asked for participant’s demographic data, and whether they feel informed about COVID-19. We have provided various web links with additional information should they wish to use them, but we also asked them whether they need more information. They were also asked to complete two standardized questionnaires that assessed their generalized anxiety disorder and depressive symptoms. The survey was provided in both the Greek and English language to increase the response rate. In order to ensure the quality of the collected data, any incomplete questionnaires were excluded from the analysis. Finally, a total of 216 participants who completed the questionnaires were included in the analysis.

### Ethical statement

The web-based questionnaire was completely voluntary and non-commercial. This study was approved by the National Bioethics Committee of Cyprus. Participants were anonymous, and they were informed regarding the purpose of the study as well as their ability to withdraw from the survey at any moment without providing any justification by having no mandatory questions.

### Measurements

#### Demographics

Demographic variables included gender, age, geographical location and occupation. For the location variable, the participants had the options of the five geographical administrative divisions found in the Republic’s non-occupied part. The occupation variable was left as free text, and after the results were collected, the authors have grouped them based on the employment sector and frequency. The categories are Healthcare, which included doctors, nurses, pharmacists, and health-related administrators; Finance, which included banking and accounting roles, Employees which include the private and public sector as well as self-employment, Students, Retired and Other.

#### Generalized anxiety disorder

We used the Greek version of the Generalized Anxiety Disorder-7 (GAD-7) scale to assess the population’s anxiety symptoms [6]. Seven items assessed the frequency of anxiety symptoms over the past two weeks on a 4-point Liker-scale ranging from 0 (never) to 3 (nearly every day). The total score of GAD-7 ranges from 0 to 21, with higher scores indicating more severe mental impairment. This questionnaire was never validated in the Cypriot population but has been shown to have a significant reliability coefficient (α = .79) in the Greek population [7]. A score above 10 is considered clinically significant, and thus we defined the presence of anxiety at a score of ≥10 [6].

#### Depression

We used the Greek translation of the Patient Health Questionnaire 9 (PHQ-9) to assess the population’s depression symptoms [8, 9]. The PHQ-9 comprises of nine items and is considered a brief, time-saving, cost-effective, easy scoring, and acceptable to patients method for both screening for depression and depression severity assessment. A major depressive disorder is considered present at a usual threshold of ≥10. This Greek version of the questionnaire was validated in the Cypriot student population and has shown a significant reliability coefficient (α = .75) [10].

### Statistical analysis

Statistical analyses included the demographic description and COVID-19-related knowledge of the Cypriot population. We also explored the prevalence of GAD and depressive symptoms stratified by gender, age, and occupation. The Chi-square test (χ2) was used to compare the differences between groups. When frequencies in categories were too small, the Fischer Exact test was used. We also performed univariate and multivariable logistic regression models to assess potential explanatory variables for GAD and major depressive symptoms during the COVID-19 outbreak. The odds ratio (OR) and 95% confidence interval (95% CI) were obtained from logistic regression models. P-values of less than 0.05 were considered statistically significant. All analyses were performed using Stata 14.

## Results

### Demographic characteristics

We have received 342 responses, out of which the ones fit for analysis were 216. Of the analyzed sample, 89 (41.20%) were males, and 104 (48.15%) were females. Ages varied from <18 to >65, and most of the respondents were in the 31-50 age group. The vast majority of the respondents were located in Nicosia (77.31%). There were various occupations, the majority being in the finance sector (31.94%). The characteristics of participants are shown in Table 1. 

**Table 1:** Demographics of mental health Covid-19 study participants.

Variable		N (%)
**Gender**	**Male**	89 (41.2)
	**Female**	104 (48.15)
	**Prefer not to state**	23 (10.65)
**Age groups**	**≤ 21**	23 (10.65)
	**22-30**	8 (3.70)
	**31-50**	118 (54.63)
	**51-65**	39 (18.06)
	**≥ 65**	9 (4.17)
	**Prefer not to state**	19 (8.80)
**Location**	**Nicosia**	167 (77.31)
	**Other (Ammochostos, Limassol, Larnaca, Paphos)**	49 (22.69)
**Occupation**	**Healthcare**	15 (6.94)
	**Finance**	69 (31.94)
	**Employee (public/private/self)**	67 (31.02)
	**Student**	23 (10.65)
	**Retired**	9 (4.17)
	**Other**	33 (15.28)

### Knowledge and information about Covid19

From the 216 participants of the study, 24% had no questions regarding COVID-19, 34% were satisfied with the information we provided, 25% wanted more general information about the disease, and 17% wanted more specific information on how to reduce the chances of contacting COVID-19 in the community as well as in the workplace (Table 2).

**Table 2:** Demographics vs. participants’ information needs.

Variable		No questions	Satisfied with information provided	General info	How to reduce chances of contracting the virus
**Gender**	**Male**	23.60	40.45	16.85	19.10
	**Female**	21.15	30.77	31.73	16.35
	**Prefer not to state**	39.13	21.74	26.09	13.04
**Age groups**	**≤ 21**	13.04	26.09	47.83	13.04
	**22-64**	21.82	36.36	22.42	19.39
	**≥≥ 65**	44.44	33.33	11.11	11.11
	**Prefer not to state**	47.37	21.05	26.32	5.26
**Occupation**	Healthcare	60.00	26.67	6.67	6.67
	**Finance**	17.39	33.33	30.43	18.84
	**Employee (public/private/self)**	20.90	43.28	17.91	17.91
	**Student**	13.04	26.09	47.83	13.04
	**Retired**	22.22	33.33	22.22	22.22
	**Other**	36.36	24.24	21.21	18.18
**Total**		24.07	33.80	25.00	17.13

### Prevalence of GAD and depression

The overall prevalence of GAD and major depressive symptoms were 13.89% and 8.33%, respectively. Table 3 shows the prevalence of GAD and major depressive symptoms by various demographic variables and their statistical significance.

**Table 3:** Prevalence of GAD and depression symptoms by demographics.

Variable		GAD No (%)	GAD Yes (%)	X_2_/Fisher (P value)	Major Depressive Symptoms No (%)	Major Depressive Symptoms Yes (%)	X^2^/Fisher (P value)
**Gender**	**Male**	88.76	11.24	1.426	89.89	10.11	0.121
	**Female**	82.69	17.31	(0.233)	91.35	8.65	(0.728)
**Age**	**≤ 21**	86.96	13.04	0.634	78.26	21.74	0.092
	**22-64**	84.24	15.76		92.12	7.88	
	**≥ 65**	100			100		
**Occupation**	**Healthcare**	86.67	13.33	0.735	93.33	6.67	0.324
	**Finance**	81.16	18.84		94.20	5.80	
	**Employee**	86.57	13.43		89.55	10.45	
	**Student**	86.96	13.04		78.26	21.74	
	**Retired**	100	0		100		
	**Other**	90.91	9.09		92.86	7.14	
**Total**		86.11	13.89		91.67	8.33	

There was a higher prevalence of GAD in females (17.31%) compared to males (11.24%). No participants aged above 65 years of age have reported major GAD. Compared with other occupational groups, people working in the finance sector reported the highest prevalence of major GAD (18.84%). There was no statistically significant difference in the prevalence of GAD and gender (p-value = 0.233), GAD, age (p-value = 0.634) or occupation (p-value = 0.735).

For major depressive symptoms, there was a higher prevalence in males (10.11%) compared to 8.65% in females. The youngest age group (≤21) had the highest prevalence (21.74%) of major depressive symptoms, and the participants >65 did not have any. The higher prevalence of major depressive symptoms of participants aged ≤21, is also represented by the student occupation group. They, have a higher prevalence of major depressive symptoms compared to other occupation groups. None of the relationships were statistically significant (p-values > 0.05) in either the χ2 or Fischer exact tests.

### Association of explanatory variables with GAD and depression during the COVID-19 outbreak

Univariate analysis of potential explanatory variables with GAD and depressive symptoms is presented in Table 4. No explanatory variable was found to have statistical significance in increasing or decreasing the odds for GAD when compared to the respective reference group. On the contrary, age has shown to affect the major depressive symptoms. More specifically, when comparing the younger age group (≤21) with the 22-64 age group, there are increased odds (3.25, 95%CI 1.04-10.17) that this age group will have depressive symptoms, and this finding is statistically significant (P< 0.05). Also, a statistically significant finding in the univariate analysis was the increased odds ratio (4.51, 95%CI 1.10-18.57) of students when compared to the Finance sector employees.

**Table 4:** Univariate analysis on GAD and Depressive symptoms.

Variable		GAD OR	95% CI	Depression OR	95% CI
Gender	**Male**	**Ref**			
	**Female**	1.65	0.72 - 3.80	0.84	0.32 - 2.22
**Age**	**22-64**	Ref			
	**≤ 21**	0.80	0.22 - 2.89	3.25	1.04 - 10.17
**Occupation**	**Finance**	Ref			
	**Healthcare**	0.66	0.13 - 3.30	1.16	0.12 - 11.19
	**Employee**	0.67	0.26 - 1.69	1.90	0.53 - 6.80
	**Student**	0.65	0.17 - 2.51	4.51	1.10 - 18.57

The multivariable logistic regression trying to include all demographics to look for associations between GAD and major depressive symptoms while adjusting for other explanatory variables found the same results observed during the univariate analysis but with mostly weakened odds ratio and the same statistical significance (Table 5). The most prominent finding from the multivariable analysis is that the odds of major depressive symptoms are increased with statistical significance to 4.91 (95%CI, 1.18-20.48) when the ≤21 age group is compared to the 22-65 age group when all other variables are kept constant.

**Table 5:** Multivariable analysis of GAD and depressive symptoms.

Variable		GAD OR	95% CI	Depression OR	95% CI
**Gender**	**Male**	**Ref**			
	**Female**	1.65	0.67 - 3.87	0.70	0.25 - 1.96
**Age**	**22-64**	Ref			
	**≤ 21**	0.46	0.09 - 2.22	4.91	1.18 - 20.48
**Occupation**	**Finance**	**Ref**			
	**Healthcare**	0.76	0.15 - 3.85	1.16	0.12 -11.37
	**Employee**	0.71	0.26 – 1.69	1.90	0.55 - 7.13

## Discussion

The Cyprus government has taken rigid measures to avoid the spread of the outbreak, without mitigating for any psychosocial effects they may incur on the population. Our study analyzed the prevalence of GAD and major depressive symptoms of the population by stratifying demographic characteristics and explored potential associations between them. This study is the first to occur in Cyprus, which can provide evidence for potential interventions that may target the vulnerable groups.

Our web-based study shows a relatively high prevalence of GAD and depressive symptoms in the Cypriot population during the COVID-19 outbreak. The prevalence of GAD and depressive symptoms may be lower than what is reported in the literature for other countries, as the numbers of infections and deaths have remained relatively low, which may be attributed to the strict measures taken early by the government [11–15].

Using the collected data, we could not identify any association between anxiety and depressive symptoms with most of the demographics we collected. We were only able to detect a statistically significant increased probability of having major depressive symptoms in students and people aged ≤21. This finding is in line with the findings found in the literature [16]. The fact that depressive symptoms were only identified in that age group/ occupation (students) could be attributed to the fact that schools closed, and this fact destabilized the students from their regular school routine. Another reason could be that some students being graduates of either high school or undergraduate university programs are now facing an uncertain future of what they will be doing in the near future: finding a job, enroll at a university in a foreign country, or further postgraduate studies. A third possible reason is the fact that a significant group of the population of Cyprus has developed resilience from the war in 1974, as well as the catastrophic effects of the 2013 economic crisis.

From our data, we saw that 42.13 of the study participants needed more information regarding COVID-19. This is an important finding as it implies that there is a big percentage of the population that has no clear picture of the situation, and there is a gap in the information reaching them, or they believe the information they get is not enough. This can inform the appropriate bodies of altering their information dissemination strategies to reach the wider population.

Some limitations that our study faced were that there might be a selection bias because we used a web-based survey to avoid possible infections as it was not allowed to meet other people other than for work-related activities. This also could be the reason why we did not have more of the older population represented as most of them are not technologically active (in 2017, it was shown that only 23% of the elderly used the Internet once a week compared to the EU average of 41%) [17]. Sampling was only based on word of mouth and was voluntary, leading to small numbers. As the study had a cross-sectional design, causal inferences cannot be made. A before and after study would be able to provide some useful insights, enabling us to compare the potential change in the mental health of the population. However, due to the unexpected trajectory or the situation, such a study was not possible.

This study can be considered as the groundwork for further investigation of the population’s mental health status as the measures that have been implemented were extended over a period of two months. A greater psychological impact is expected to appear in the future as it is still too early in predicting the additional economic effect the pandemic will have.

## Conclusion

We identified a mental health burden of the public during the COVID-19 outbreak, with mostly young people being affected. Our study findings can provide data support for targeted interventions on the psychological health of the population during the outbreak. Surveillance of the psychosocial impact the pandemic may have on the population is of paramount importance as these effects will have an impact on not just the healthcare system, but it will be a systemic problem. Establishing early interventions to tackle those effects should be part of any preparedness plan for such situations.

## Conflict of Interest

The authors declare that there is no conflict of interest.

## Acknowledgment

The authors would like to acknowledge EY and SAP for their assistance in setting up the survey.
